# GCKR Polymorphisms Increase the Risks of Low Bone Mineral Density in Young and Non‐Obese Patients With MASLD and Hyperuricemia

**DOI:** 10.1002/kjm2.70017

**Published:** 2025-04-09

**Authors:** Tzu‐Hao Li, Yu‐Shin Huang, Chia‐Chen Ma, Shin‐Yu Tsai, Hung‐Cheng Tsai, Hsiao‐Yun Yeh, Hsiao‐Chin Shen, Shiao‐Ya Hong, Chien‐Wei Su, Hwai‐I Yang, Ying‐Ying Yang, Ming‐Chih Hou

**Affiliations:** ^1^ Division of Allergy, Immunology, and Rheumatology, Department of Internal Medicine Shin Kong Wu Ho‐Su Memorial Hospital Taipei Taiwan; ^2^ Institute of Clinical Medicine National Yang Ming Chiao Tung University Taipei Taiwan; ^3^ School of Medicine College of Medicine, National Yang Ming Chiao Tung University Taipei Taiwan; ^4^ School of Medicine Fu Jen Catholic University New Taipei City Taiwan; ^5^ Department of Medical Education Taipei Veterans General Hospital Taipei Taiwan; ^6^ Division of Allergy, Immunology, and Rheumatology, Department of Internal Medicine Taipei Veteran General Hospital Taipei Taiwan; ^7^ Department of Chest Medicine Taipei Veterans General Hospital Taipei Taiwan; ^8^ Department of Biotechnology and Laboratory Science in Medicine National Yang Ming Chiao Tung University Taipei Taiwan; ^9^ Department of Internal Medicine, Division of Gastroenterology and Hepatology Taipei Veterans General Hospital Taipei Taiwan; ^10^ Genomics Research Center Academia Sinica Taipei Taiwan

**Keywords:** hyperuricemia, low bone mineral density, metabolic‐associated steatotic liver disease, single nucleotide polymorphisms

## Abstract

Metabolic‐associated steatotic liver disease (MASLD) encompasses common comorbidities including low bone mineral density (BMD) and hyperuricemia (HU), yet relevant genetic analyses are limited. This study aimed to investigate the genetic effects of risk single nucleotide polymorphisms (SNPs) on the occurrence of low BMD in patients with MASLD and HU, particularly focusing on relatively young or non‐obese populations. We conducted a cross‐sectional study utilizing data from the Taiwan Biobank, screening a total of 150,709 participants who were prospectively enrolled over a period of 13 years. The risk SNPs for MASLD were identified. Genotype analyses of HU and its effects on the occurrence of low BMD in the general population were evaluated, with further analyses of common SNPs focusing on patients with MASLD, including subgroup analyses on relatively young and non‐obese populations. A total of 20,496 participants were eligible for analysis, including 7526 patients with MASLD. Several risk SNPs for MASLD were identified. Furthermore, MASLD patients carrying the PNPLA3‐rs738409 C_C, PNPLA3‐rs2896019 T_T, GCKR‐rs780094 T_T, and GCKR‐rs1260326 T_T genotypes exhibited an increased risk of comorbidity with HU. Trend analysis revealed that the T alleles in GCKR‐rs780094 and GCKR‐rs1260326 were associated with the occurrence of low BMD in MASLD individuals comorbid with HU, particularly among relatively young or non‐obese populations. In relatively young, non‐obese patients with MASLD and HU, genetic effects significantly increase the risk of occurrence of low BMD. Given the presence of genetic effects in these ostensibly low‐risk groups, heightened awareness and close follow‐up are recommended.

## 1 Introduction

Metabolic‐associated steatotic liver disease (MASLD) represents the intersection of cardiovascular and metabolic disorders with hepatic steatosis, and substantial evidence indicates the close association with the progression of MASLD and increased risk of not only hepatic complications, but also other systemic comorbidities including cardiovascular diseases (CVDs) and fractures with subsequent mortality [1]. Hence, investigations into risk single‐nucleotide polymorphisms (SNPs) in MASLD, including the hepatic comorbidities and CVDs, have been conducted, but predominantly within Caucasian populations [2]. Therefore, comprehensive genetic analyses focusing on MASLD and other comorbidities within Asian populations are warranted.

Hyperuricemia (HU) is a prevalent comorbidity associated with MASLD, as both conditions can mutually exacerbate and worsen the cardiometabolic burden [3]. Furthermore, HU is associated with increased rates of fibrosis and heightened mortality from CVDs in patients diagnosed with MASLD [2, 4]. Although HU is typically more prevalent among the elderly, genetic factors may present significant risks and play critical roles in the development of concurrent metabolic disorders in non‐elderly individuals [5]. Furthermore, although being overweight has traditionally been considered protective against bone loss, accumulating studies have elucidated the risk of osteoporosis in patients with MASLD even in those with obesity [1]. In fact, the detrimental effects of MASLD on bone health can lead to low bone mineral density (BMD) even at relatively young ages [6]. Despite the significance of these comorbidities in MASLD, limited information has focused on the SNPs associated with MASLD comorbid with low BMD and HU.

The interrelationship between HU and low BMD remains controversial. In patients with MASLD, HU and low BMD frequently coexist as comorbidities, potentially linked through common pathways involving insulin resistance (IR) and chronic inflammation [1, 7]. Additionally, in young and non‐obese populations, HU is highly associated with metabolic syndrome [8]; similarly, osteoporosis in young and non‐obese individuals is also associated with metabolic syndrome and IR [9, 10]. Consequently, the identification of relevant risk SNP genotypes potentially associated with IR and chronic inflammation, along with the effects in young and non‐obese patients with MASLD, warrants further investigation, despite the current limited literature on this topic.

Taiwan Biobank (TWB) is a government‐supported, large‐scale nationwide database that contains extensive epidemiological and biomedical information, including DNA profiles, laboratory data, and imaging reports. As of August 2021, the database had enrolled over 150,000 individuals [11]. Utilizing data from TWB, we aimed to evaluate the genetic effects on the occurrence of MASLD and its comorbidities, including HU and low BMD, especially among low‐risk individuals, such as relatively young and non‐obese populations.

## 2 Methods

### 2.1 Database Source and Study Participants

The cross‐sectional observational study utilized data from the TWB. Informed consent was obtained in writing during participant recruitment, thereby facilitating the collection and sharing of anonymized data. The detailed methodology has been previously reported [11]. The participants predominantly belong to Han Chinese ancestry, which includes Hokkien, Hakka, Mainlander, and Indigenous groups, thus reflecting the demographic composition of Taiwan.

Among the participants, those with missing data for any of the cardiometabolic criteria essential for the diagnosis of MASLD were excluded. The criteria included: (1) waist circumference (WC), (2) serum glycated hemoglobin (HbA1c) and glucose levels, (3) serum triglycerides (TG), (4) serum high‐density lipoprotein cholesterol (HDL‐C), and (5) blood pressure. Additionally, participants without an abdominal ultrasound report were also excluded from the analysis. Eligible participants were subsequently categorized into MASLD and non‐MASLD groups based on the current diagnostic criteria for MASLD [12]. This study was performed in accordance with the Declaration of Helsinki and received approval from the Institutional Ethical Review Board of Academia Sinica (AS‐IRB‐BM‐16015).

### 2.2 Genetic Analysis

The TWB has developed two SNP arrays for genetic research: TWBv1 and TWBv2. To enhance the utility of these resources, we integrated imputation data from TWBv1 and TWBv2, following rigorous pre‐imputation quality control (QC) to exclude variants and samples with high missing data. Genetic ancestry inference and population outlier detection were performed using principal component analysis, referencing the 1000 Genomes Project. During the East Asian QC phase, low call rate variants and those failing Hardy–Weinberg equilibrium were removed. TWB genotypes were subsequently phased and imputed utilizing the 1KG EAS panel, followed by post‐imputation QC to filter out poorly imputed variants.

### 2.3 Data Collection

For the participants in TWB, a trained nurse administered a structured questionnaire that encompassed the participants' medical history, as well as their smoking and alcohol consumption habits. Additionally, relevant demographic, clinical, and basic biochemical data were collected, along with other variables pertinent to MASLD. HU was defined as serum uric acid (UA) levels exceeding 7 mg/dL in men and 6 mg/dL in women [5]. DNA and blood samples were also collected in a clinically appropriate setting.

Abdominal ultrasonography was conducted using a standard ultrasound system operated by experienced technicians. The diagnosis of hepatic steatosis was established in accordance with the guidelines, including: (1) an increased liver echogenicity relative to the kidneys, (2) a diffuse hyperechoic echotexture, (3) deep attenuation, and (4) vascular blurring, and the reliability and accuracy of it have been previously documented.

The BMD of participants at the femoral site was assessed using either dual‐energy X‐ray absorptiometry (DEXA) scans (Hologic, Bedford, MA, USA) or quantitative ultrasound at heels (qUS, Achilles InSight, GE); the BMD measurements using these two modalities were highly correlated, as previously mentioned [13]. Low BMD was defined as a T‐score of less than −1, which encompasses osteopenia (−1 < T‐score ≦ −2.5) and osteoporosis (T‐score < −2.5).

### 2.4 Definition of MASLD and Various Calculated Metabolic Indices

As the previously mentioned, in the presence of hepatic steatosis, if at least one of the cardiometabolic risk factors (CMRFs) exists, MASLD could be diagnosed. The CMRFs include: (1) body mass index (BMI) ≥ 23 kg/m^2^ or WC > 90 cm in men, > 80 cm in women, (2) fasting serum glucose ≥ 100 mg/dL or 2‐h post‐load glucose level ≥ 140 mg/dL or HbA1c ≥ 5.7% or the use of specific drug treatment, (3) blood pressure ≥ 130/85 mmHg or the use of specific drug treatment, (4) plasma TG ≥ 150 mg/dL or the use of specific drug treatment, (5) plasma HDL‐C < 40 mg/dL for men and < 50 mg/dL for women or the use of specific drug treatment [12].

Utilizing anthropometric and biochemical data, various indicators of adiposity were computed, including BMI and waist‐to‐hip ratio (WHR). The BMI (expressed in kg/m^2^) is determined by dividing body weight (kg) by the square of height (in meters). WHR is computed with the formula: WHR = WC (cm)/HC (cm). Moreover, the triglyceride‐glucose (TyG) index is recognized as an easily accessible biomarker for assessing IR. It is calculated using the following formula: TyG = Ln (fasting TG [mg/dL] × fasting glucose [mg/dL]/2). Furthermore, two additional markers were employed for the assessment of IR: the TyG‐BMI index, which is derived from the formula: BMI (kg/m^2^) × TyG index, and the TyG‐WHR index, which was computed as: TyG index × WHR. Also, the ratio of TG to HDL‐C (TG/HDL‐C ratio) was calculated as TG divided by HDL‐C, and was another convenient tool for detecting IR and chronic inflammation [14, 15].

### 2.5 Statistical Analysis

In the current investigation, Student's *t* test and Pearson's chi‐square were utilized for the analysis of continuous and categorical variables, respectively. Differences between genotypes and odds ratios (ORs) with 95% confidence intervals (CIs) were analyzed using linear and logistic models adjusted for multiple comparison test with Bonferroni method, to avoid type I error [16]. The most prevalent genotype among the candidate SNPs was designated as the reference for analyses of association with clinical outcomes, thereby ensuring stable and dependable OR estimates while enhancing the statistical integrity of our results. The Cochran‐Armitage trend test was employed to evaluate the linear trend in per‐allele ORs of various candidate genotypes concerning the risk of clinical outcomes. To rule out the confounding effects of other variables on decreased BMD, the association analysis was adjusted by sex, menopause, and smoking with the use of logistic regression analysis. Statistical analyses were conducted using IBM SPSS Statistics, version 25.0 (IBM Corp., Armonk, NY, USA).

## 3 Results

### 3.1 Patient Characteristics

**FIGURE 1:**
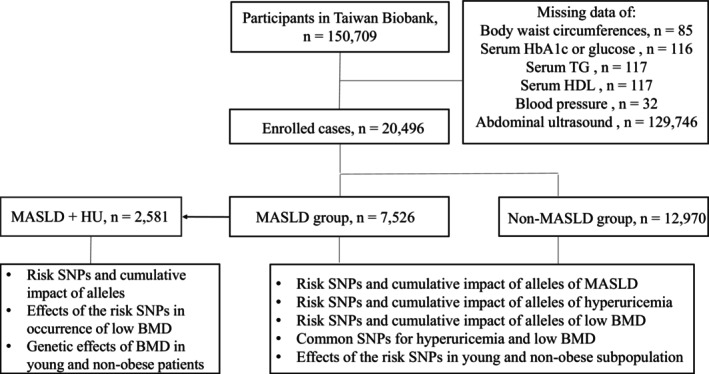
Flow chart of our study. In addition to dividing the subjects into MASLD and non‐MASLD groups, we further extracted a MASLD subgroup comorbid with HU for the analysis of low BMD‐associated SNPs. BMD, bone mineral density; HbA1c, hemoglobin A1c; HDL, high‐density lipoprotein cholesterol; HU, hyperuricemia; MASLD, metabolic dysfunction‐associated steatotic liver disease; SNP, single nucleotide polymorphism; TG, triglyceride.

Between December 2008 and November 2021, 150,709 participants were recruited from the TWB. As shown in Figure 1, after the exclusion of patients without adequate data, a total of 20,496 participants were included in the final analysis, among whom 7526 were diagnosed with MASLD. Among patients with MASLD, 1609, 2229, 2091, 1222, and 375 participants had 1, 2, 3, 4, and 5 CMRFs, respectively. In addition, 4453, 2480, and 593 participants had mild, moderate, and severe steatosis, respectively.

The characteristics of the study population are summarized in Table S1, and the proportions of co‐existed etiologies including alcohol consumption and viral hepatitis were also exhibited. Patients with MASLD had higher blood pressure, fasting blood glucose, HbA1c, TG, and TG/HDL‐C ratio, along with lower HDL‐C and elevated serum UA. In terms of metabolic markers, MASLD patients also had increased BMI, WHR, and IR, as indicated by a higher TyG, TyG‐BMI, and TyG‐WHR indices. Additionally, the higher chronic inflammatory marker, TG/HDL ratio, was associated with high UA level and the proportion of HU in MASLD patients (Table S1).

### 3.2 Association Between Genotypes and MASLD

The distribution of genotypes between the MASLD and non‐MASLD groups is detailed in Table S2. Participants carrying the PNPLA3‐rs738409 G_G, PNPLA3‐rs2896019 G_G, GCKR‐rs780094 T_T, GCKR‐rs1260326 T_T, and TM6SF2‐rs58542926 C_T or T_T genotypes were at increased risk of MASLD. Trend analysis further confirmed that the T allele in APOE‐rs429358, GCKR‐rs780094, GCKR‐rs1260326, and TM6SF2‐rs58542926, as well as the G allele in PNPLA3‐rs738409 and PNPLA3‐rs2896019, were significant risk factors for MASLD (Table S2). Among these SNPs, the proportions of participants carrying GCKR‐rs780094 T_T and GCKR‐rs1260326 T_T were higher in Hokkien than in other subpopulations, and that of other SNPs did not differ significantly among the various subpopulations.

### 3.3 Association Between Genotypes and Hyperuricemia in General Population, and in Non‐Obese and Relatively Young Subgroups

The association between specific SNP genotypes and HU is detailed in Table S3. Participants carrying the PNPLA3‐rs738409 C_C, PNPLA3‐rs2896019 T_T, GCKR‐rs780094 T_T, and GCKR‐rs1260326 T_T genotypes were at higher risk of HU. Trend analysis further identified the T allele in PNPLA3‐rs2896019, GCKR‐rs780094, and GCKR‐rs1260326 as a significant risk factor for HU.

The association between specific SNP genotypes and HU in the non‐obese population by WHO definition (BMI < 30) was shown in Table 1. Non‐obese individuals carrying the previously mentioned risk alleles at PNPLA3‐rs738409 C_C, PNPLA3‐rs2896019 T_T, GCKR‐rs780094 T_T, and GCKR‐rs1260326 T_T were still at high risk of HU. Trend analysis further indicated that the T allele in PNPLA3‐rs2896019, GCKR‐rs780094, and GCKR‐rs1260326 was a significant risk factor for HU. For the obese population, there were no similar trends of risk alleles in these SNPs (Table S4).

**TABLE 1:** Associated SNPs of hyperuricemia in the non‐obese general population.

SNP	Genotype	HU	Non‐HU	%	Odds ratio	95% CI	*p*
PNPLA3‐rs738409							
Risk allele: C	C_C	1517	5436	21.8	1.149	1.063–1.242	< 0.001
	C_G	1704	7018	19.5	Reference		
	G_G	526	2203	19.3	0.983	0.882–1.096	0.763
	Trend						0.001
PNPLA3‐rs2896019							
Risk allele: T	G_G	551	2337	19.1	0.830	0.747–0.923	0.001
	G_T	1718	7087	19.5	Reference		
	T_T	1476	5224	22.0	1.166	1.078–1.260	0.001
	Trend						0.001
GCKR‐rs780094							
Risk allele: T	C_C	871	4019	17.8	0.839	0.768–0.917	< 0.001
	C_T	1897	7344	20.5	Reference		
	T_T	979	3294	22.9	1.151	1.054–1.256	0.002
	Trend						< 0.001
GCKR‐rs1260326							
Risk allele: T	C_C	825	3818	17.8	0.843	0.770–0.923	< 0.001
	C_T	1892	7383	20.4	Reference		
	T_T	1030	3456	23.0	1.163	1.067–1.267	0.001
	Trend						< 0.001

*Note*: Categorical data are expressed as the number of patients. The *p* value is analyzed by logistic regression. Reference was designated based on the most prevalent genotype.

Abbreviations: BMD, bone mineral density; CI, confidence interval; GCKR, glucokinase regulator; HU, hyperuricemia; PNPLA3, patatin like phospholipase domain containing 3; SNP, single nucleotide polymorphism.

In the relatively young population (age < 60, as previously defined [17]), the association between specific SNP genotypes and HU is presented in Table 2. Participants carrying the GCKR‐rs780094 T_T and GCKR‐rs1260326 T_T genotypes were at higher risk of HU. Trend analysis further identified the T alleles in GCKR‐rs780094 and GCKR‐rs1260326 as significant risk factors for HU in the relatively younger population. In contrast, no similar trends in risk alleles were observed in the elderly population (Table S5).

**TABLE 2:** Associated SNPs of hyperuricemia in the relatively young general population.

SNP	Genotype	HU	Non‐HU	%	Odds ratio	95% CI	*p*
GCKR‐rs780094							
Risk allele: T	C_C	697	3174	18.0	0.823	0.745–0.909	< 0.001
	C_T	1525	5715	21.1	Reference		
	T_T	784	2580	23.3	1.136	1.030–1.253	0.011
	Trend						< 0.001
GCKR‐rs1260326							
Risk allele: T	C_C	657	3013	17.9	0.822	0.742–0.910	< 0.001
	C_T	1525	5747	21.0	Reference		
	T_T	824	2709	23.3	1.146	1.041–1.262	0.005
	Trend						< 0.001

*Note*: Categorical data are expressed as the number of patients. The *p* value is analyzed by logistic regression. Reference was designated based on the most prevalent genotype.

Abbreviations: BMD, bone mineral density; CI, confidence interval; GCKR, glucokinase regulator; HU, hyperuricemia; SNP, single nucleotide polymorphism.

### 3.4 Association Between Genotypes and Low BMD in General Population and Analyses of Genetic Effects of Common SNPs in Hyperuricemia and Low BMD

**FIGURE 2:**
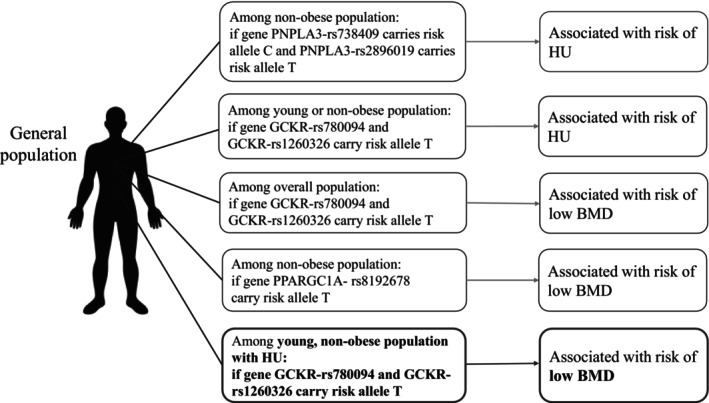
Genetic associations with hyperuricemia and low BMD in non‐obese and relatively young general populations. PNPLA3‐rs738409 carrying risk allele C and PNPLA3‐rs2896019 carrying risk allele T, as well as GCKR‐rs780094 and GCKR‐rs1260326 with the T allele, are identified as risk factors for HU in the non‐obese population. In the young population, the GCKR‐rs780094 and GCKR‐rs1260326 variants carrying the T allele increase the risk of HU. In the general population, the PPARGC1A‐rs8192678 variant with the T allele is associated with an increased risk of low BMD. On the other hand, among non‐obese individuals, the PPARGC1A‐rs8192678 T allele also elevates the risk of low BMD. In young, non‐obese individuals with HU, the T allele in GCKR‐rs780094 and GCKR‐rs1260326 is linked to a higher risk of low BMD. BMD, bone mineral density; GCKR, glucokinase regulator; HU, hyperuricemia; MASLD, metabolic dysfunction‐associated steatotic liver disease; PNPLA3, patatin‐like phospholipase domain‐containing protein 3; PPARGC1A, peroxisome proliferator‐activated receptor γ–coactivator 1α.

Participants with the GCKR‐rs780094 T_T, GCKR‐rs1260326 T_T, and PPARGC1A‐rs8192678 T_T genotypes exhibited a higher risk of low BMD, and the risk effects of the corresponding T alleles were unveiled (Table S6). GCKR‐rs780094 T_T and GCKR‐rs1260326 T_T also exhibited a higher risk of low BMD in both relatively young patients and non‐obese patients with HU, and further trend analysis demonstrated the effects of low BMD resulting from the presence of the T risk alleles (Tables S7 and S8). Additionally, the markers of IR, TyG‐WHR, were significantly higher in young or non‐obese individuals with low BMD (Tables S7 and S8). The genetic effects in the relatively young and non‐obese patients with HU and low BMD are graphically represented in Figure 2.

### 3.5 Association Between Risk Genotypes and Hyperuricemia as Well as Low BMD in MASLD, and the Genetic Effects of Common Risk SNPs in GCKR

Patients with MASLD carrying the PNPLA3‐rs738409 C_C, PNPLA3‐rs2896019 T_T, GCKR‐rs780094 T_T, and GCKR‐rs1260326 T_T showed an increased risk of HU, and trend analysis further demonstrated the roles of the T alleles as significant risk factors for low BMD (Table 3). Additionally, the marker of IR, TyG‐WHR, was significantly higher in MASLD patients with low BMD (Table 3). Notably, in the patients with fewer CMRFs (1 or 2 CMRFs) comorbid with HU, the PNPLA3‐rs2896019 T_T emerged as the predominant SNP, and the trend analysis demonstrated the risk of T alleles (Table S9). Concerning the occurrence of low BMD, the patients with MASLD carrying the GCKR‐rs780094 T_T and GCKR‐rs1260326 T_T had an increased risk of low BMD, and the T alleles among these two SNPs are risk alleles for low BMD (Table 3).

**TABLE 3:** The insulin resistance marker and associated SNPs of MASLD with hyperuricemia or low BMD.

SNP	Genotype	HU	Non‐HU	%	Odds ratio	95% CI	*p*
MASLD with HU							
TyG‐WHR	—	7.64 ± 0.76	7.52 ± 0.75	—	—	—	< 0.001
PNPLA3‐rs738409							
Risk allele: C	C_C	941	1551	37.8	1.160	1.041–1.292	0.007
	C_G	1156	2210	34.3	Reference		
	G_G	420	888	32.1	0.984	0.789–1.036	0.147
	Trend						0.002
PNPLA3‐rs2896019							
Risk: T	G_G	435	923	32.0	0.913	0.708–1.044	0.184
	G_T	1151	2230	34.0	Reference		
	T_T	928	1493	38.3	1.205	1.080–1.342	0.001
	Trend						0.001
GCKR‐rs780094							
Risk allele: T	C_C	587	1235	32.2	0.896	0.794–1.010	0.071
	C_T	1243	2342	34.7	Reference		
	T_T	687	1072	39.1	1.208	1.073–1.359	0.002
	Trend						< 0.001
GCKR‐rs1260326							
Risk allele: T	C_C	547	1164	32.0	0.880	0.779–0.995	0.041
	C_T	1250	2434	33.9	Reference		
	T_T	720	1144	38.6	1.179	1.049–1.322	0.005
	Trend						0.001
SNP	Genotype	Low BMD	Normal BMD	%	Odds ratio	95% CI	*p*
MASLD with low BMD							
TyG‐WHR	—	7.62 ± 0.74	7.52 ± 0.76	—	—	—	< 0.001
GCKR‐rs780094							
Risk allele: T	C_C	703	1148	37.9	0.878	0.783–0.985	0.026
	C_T	1502	2154	41.0	Reference		
	T_T	768	1036	42.6	1.063	0.949–1.192	0.294
	Trend						0.036
GCKR‐rs1260326							
Risk allele: T	C_C	665	1075	38.2	0.902	0.802–1.014	0.083
	C_T	1488	2169	40.7	Reference		
	T_T	820	1049	43.9	1.139	1.018–1.276	0.023
	Trend						0.040

*Note*: Continuous data are expressed as level ± standard deviation. Categorical data are expressed as number of patients. *p* value is analyzed by logistic regression. Reference was designated based on the most prevalent genotype.

Abbreviations: BMD, bone mineral density; CI, confidence interval; GCKR, glucokinase regulator; HU, hyperuricemia; PNPLA3, patatin‐like phospholipase domain containing 3; SNP, single nucleotide polymorphism; TyG‐WHR, triglyceride‐glucose index‐waist to height ratio.

**FIGURE 3:**
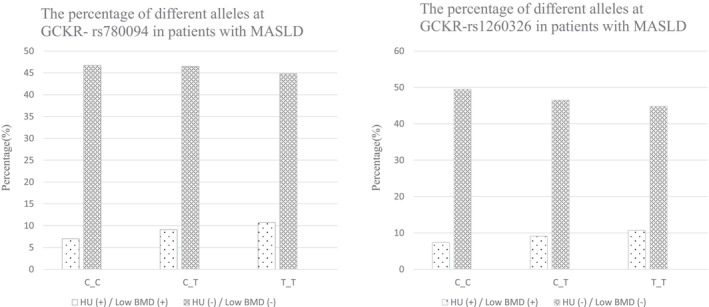
Concurrent hyperuricemia and low BMD in MASLD with the cumulative effects of the T alleles in common SNPs. In patients with MASLD, the prevalence of concurrent HU and low BMD increases with the number of T alleles in GCKR‐rs780094 and GCKR‐rs1260326. BMD, bone mineral density; GCKR, glucokinase regulator; HU, hyperuricemia; MASLD, metabolic dysfunction‐associated steatotic liver disease; SNPs, single nucleotide polymorphisms.

**FIGURE 4:**
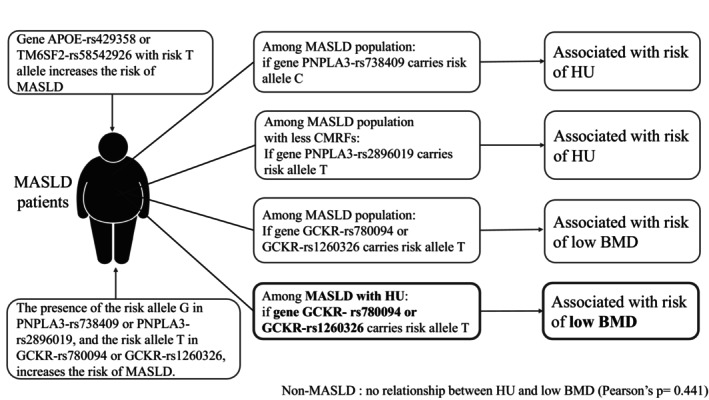
Conclusion of risk SNPs of hyperuricemia and low BMD in MASLD patients. The T allele in APOE‐rs429358 or TM6SF2‐rs58542926 increases the risk of MASLD; meanwhile, the G allele in PNPLA3‐rs738409 or PNPLA3‐rs2896019, and the T allele in GCKR‐rs780094 or GCKR‐rs1260326 also contribute to an elevated risk of MASLD. In the MASLD population, the PNPLA3‐rs738409 variant with the C risk allele is associated with an increased risk of HU. Among MASLD individuals with fewer CMRFs, the PNPLA3‐rs2896019 variant carrying the T risk allele is linked to a higher risk of HU. In the MASLD population, the GCKR‐rs780094 and GCKR‐rs1260326 variants, both carrying the T risk allele, are associated with an elevated risk of low BMD. Among MASLD patients comorbid with HU, the T risk allele in both GCKR‐rs780094 and GCKR‐rs1260326 is similarly associated with an increased risk of low BMD. For the non‐MASLD population, there is no relationship between HU and low BMD. APOE, apolipoprotein E; BMD, bone mineral density; CMRF, cardiometabolic risk factor; GCKR, glucokinase regulator; HU, hyperuricemia; MASLD, metabolic dysfunction‐associated steatotic liver disease; PNPLA3, patatin‐like phospholipase domain containing 3; PPARGC1A, peroxisome proliferator‐activated receptor γ coactivator 1α; SNP, single nucleotide polymorphism; TM6SF2, transmembrane 6 superfamily member 2.

The previously mentioned SNPs in GCKR serve as the common risk genotypes of HU and low BMD among patients with MASLD, and the prevalence of concurrent HU and low BMD increases with the number of T alleles, as illustrated in Figure 3. In the MASLD patients comorbid with HU, individuals with the GCKR‐rs780094 T_T and the GCKR‐rs1260326 T_T demonstrated an increased risk of low BMD, and further trend analysis demonstrated the cumulative effects of T alleles in these SNPs (Table 4). To exclude the potential confounding effects of sex, menopause, and smoking, the genetic effects of T alleles in GCKR‐rs780094 and GCKR‐rs1260326 were analyzed by adjusting these variables. The results exhibited the risks of these alleles on low BMD (Table S10). This trend was also observed in both the relatively young MASLD comorbid with HU subgroup and the non‐obese MASLD comorbid with HU subgroup (Tables S11 and S12). Also, the TyG‐WHR was significantly higher in young MASLD patients with HU low BMD, as well as non‐obese MASLD patients with HU low BMD (Tables S11 and S12). The genetic effects of the previously mentioned SNPs were summarized in Figure 4.

**TABLE 4:** Associated SNPs of occurrence of low BMD in MASLD with hyperuricemia patients.

SNP	Genotype	Low BMD	Normal BMD	%	Odds ratio	95% CI	*p*
GCKR‐rs780094							
Risk allele: T	C_C	218	368	37.2	0.776	0.635–0.948	0.013
	C_T	514	728	41.4	Reference		
	T_T	306	381	44.5	1.341	1.006–1.627	0.003
	Trend						0.008
GCKR‐rs1260326							
Risk allele: T	C_C	207	339	37.9	0.816	0.665–1.000	0.050
	C_T	509	740	40.8	Reference		
	T_T	322	398	44.7	1.176	0.977–1.416	0.086
	Trend						0.013

*Note*: Categorical data are expressed as the number of patients. The *p* value is analyzed by logistic regression. Reference was designated based on the most prevalent genotype.

Abbreviations: BMD, bone mineral density; CI, confidence interval; GCKR, glucokinase regulator; HU, hyperuricemia; SNP, single nucleotide polymorphism.

## 4 Discussion

Osteoporosis is a pervasive public health issue, and its progression is irreversible, making early screening for BMD essential to prevent further deterioration, despite low adherence from healthcare practitioners [18]. Recent studies have increasingly focused on outcomes related to low BMD, defined as a T‐score < −1, rather than solely on osteoporosis, to mitigate the risk of exacerbating BMD loss in individuals with metabolic disorders [19]. Although accumulating evidence, including meta‐analyses, highlights the elevated risk of low BMD and osteoporosis along with causal associations, recommendations for screening and monitoring BMD, as well as treatment strategies aimed at MASLD, have yet to be established [20]. In the present study, we examined the genetic effects of GCKR‐rs780094 and GCKR‐rs1260326 on the occurrence of low BMD among non‐obese or relatively young patients with MASLD comorbid with HU. In addition, the impact of MASLD on decreasing BMD appears to be more pronounced in Asians compared to non‐Asians, and a higher prevalence of T alleles in GCKR‐rs780094 and GCKR‐rs1260326 has been observed in Asian populations, including Chinese and Japanese [20, 21]. Together with our findings and the greater dominance of genetic frequency and effects within the Asian population, screening for BMD should be more vigilant and proactive, particularly in relatively young and non‐obese subgroups of MASLD patients comorbid with HU.

Several studies have reported a protective effect of HU on BMD loss in postmenopausal women and general elderly individuals, regardless of sex and ethnicity [22, 23]. Conversely, other literature has indicated no significant association between HU and BMD, with some studies displaying a negative impact of HU on BMD in the general population and among postmenopausal women [24, 25]. This suggests that the effects of HU on BMD may depend on varying clinical conditions. In chronic inflammatory contexts, such as psoriasis, HU has been associated with decreased BMD and an increased risk of osteoporotic fractures [26]. These findings elucidate that HU‐induced pro‐inflammatory cytokines can activate osteoclasts and inhibit osteoblasts, ultimately resulting in BMD loss, particularly in the presence of inflammatory diseases [27].

MASLD has been recognized as a condition characterized by chronic low‐grade inflammation [28]. In non‐obese MASLD individuals, HU is an important critical trigger for chronic inflammation and the MASLD progression [29]. Particularly, in our study, the higher inflammatory marker TG/HDL ratio was associated with HU in MASLD patients [14, 15]. Among relatively young patients with MASLD, the degree of inflammation and associated comorbidities may be more severe [30]. The gene GCKR encodes the glucokinase regulatory protein, which can modulate glucokinase post‐translationally, thereby influencing glycolysis and, consequently, glycolysis‐mediated inflammation and macrophage activation [31, 32]. Additionally, the regulation of glucokinase also affects inflammation through alterations in lipolysis and β‐oxidation [33]. Previous literature has indicated an association between T alleles in GCKR‐rs780094 and GCKR‐rs1260326 and increased fasting blood sugar levels resulting from enhanced glycolysis, suggesting a potential increase in subsequent inflammation [34]. In the present study, we present additional genetic effects of GCKR on HU‐mediated low BMD in subgroups characterized by heightened inflammatory responses.

HU is associated with IR, which significantly increases the risk of BMD through IR‐induced hyperinsulinemia [35, 36]. Moreover, inflammation resulting from HU can further exacerbate IR, particularly in the context of metabolic abnormalities [37]. In non‐obese patients with MASLD, IR is predominant and associated with HU, even in the absence of other metabolic risk factors [38]. Additionally, age is negatively correlated with IR in both sexes, suggesting that IR may be more pronounced in relatively young patients with MASLD [39]. The SNPs GCKR‐rs780094 and GCKR‐rs1260326 have been associated with IR in MASLD, potentially by enhancing glucokinase activation and subsequent hepatic glucose metabolism [40]. Furthermore, T alleles in GCKR‐rs780094 and GCKR‐rs1260326 are linked to IR and metabolic syndrome [30]. Thus, IR may provide an additional explanation for the genetic effects observed in these subgroups, distinguishing them from obese and elderly patients.

The strengths of this study include its large‐scale sample of over 150,000 participants from Chinese populations, who were prospectively enrolled over a period of 13 years, to identify risk SNPs sequentially from the general population to patients with MASLD, and further analyses in relatively young and non‐obese groups. However, the limitations exist in the cross‐sectional nature of the study, which prevents the present study from establishing causal relationships between the risk SNPs and HU and low BMD. Also, the large sample size could cause false positivity resulting from the accumulation of type 1 error, and we managed to correct the results by multiple comparison tests with the Bonferroni method, which was the most conservative and common adjustment to avoid type I error [16]. Besides, the occurrence of MASLD, HU, and low BMD are complex traits influenced by lifestyle habits and environmental factors, and the interactions among genetic effects and these potentially detrimental factors could not be analyzed in the present study.

## 5 Conclusion

In conclusion, patients with T alleles in GCKR‐rs780094 and GCKR‐rs1260326 are associated with an increased risk of comorbidities involving both HU and low BMD in individuals with MASLD. These SNPs enhance the risk of occurrence of low BMD in MASLD patients comorbid with HU, particularly significant among non‐obese and relatively young individuals. Although the risk of BMD loss may seem ostensibly low due to age and anthropometric characteristics, genetic effects are still significant in this subgroup. Therefore, heightened awareness and close follow‐up are recommended.

## Conflicts of Interest

The authors declare no conflicts of interest.

## Supporting information


**Data S1.** Tables.

## Data Availability

Data sharing is not applicable to this article as no new data were created or analyzed in this study.
