# Supplementary material for: GCKR Polymorphisms Increase the Risks of Low Bone Mineral Density in Young and Non‐Obese Patients With MASLD and Hyperuricemia
Source: Kaohsiung J Med Sci. 2025 Apr 9;41(6):e70017. doi: 10.1002/kjm2.70017 (PMC12199583; doi:10.1002/kjm2.70017)
Supplement: Supplementary file 1 — Data S1. Tables. [file KJM2-41-e70017-s001.docx]

Supplementary Table S1

Demographics of participants

|  | MASLD (n=7521) | Non-MASLD (n=12975) | *p* |
| --- | --- | --- | --- |
| Demographics |  |  |  |
| Age | 52.36 ± 9.95 | 51.10 ± 10.53 | <0.001* |
| Male (n, %) | 3336 (43.36 %) | 3963 (30.54 %) | <0.001* |
| Hypertension  (>130/85) (n, %) | 3111 (41.36 %) | 3036 (23.40 %) | <0.001* |
| Alcohol consumption (n, %) | 769 (10.22 %) | 982 (7.64 %) | <0.001* |
| Hepatitis B (n, %) | 720 (9.57 %) | 1537 (12.06 %) | 0.001* |
| Hepatitis C (n, %) | 140 (1.86 %) | 305 (2.35 %) | 0.086 |
| Laboratory data |  |  |  |
| FBS>100 (n, %) | 2461 (32.72 %) | 1979 (15.25 %) | <0.001* |
| HbA1c | 5.96 ± 0.92 | 5.63 ± 0.67 | <0.001* |
| HbA1c>=5.7 (n, %) | 3899 (51.84 %) | 3457 (26.64 %) | <0.001* |
| TG>150 (n, %) | 2717 (36.13 %) | 1328 (10.24 %) | <0.001* |
| Low HDL  (M <40; F <50) (n, %) | 3066 (41.36 %) | 2406 (18.54 %) | <0.001* |
| TG/HDL | 0.18 ± 0.04 | 0.15 ± 0.04 | <0.001* |
| Uric acid | 6.03 ± 1.44 | 5.18 ± 1.31 | <0.001* |
| Hyperuricemia (n, %) | 2581 (40.77 %) | 1844 (14.21 %) | <0.001* |
| Metabolic surrogate |  |  |  |
| BMI (kg/m2) | 26.09 ± 3.51 | 22.86 ± 3.11 | <0.001* |
| WHR | 0.90 ± 0.06 | 0.85 ± 0.07 | <0.001* |
| TyG | 8.41 ± 0.62 | 8.43 ± 0.62 | 0.016* |
| TyG-BMI | 219.41 ± 33.72 | 192.69 ± 29.56 | <0.001* |
| TyG-WHR | 7.56 ± 0.75 | 7.17 ± 0.76 | <0.001* |
| Bone mineral density |  |  |  |
| T-score | -0.4620 ± 1.54 | -0.3775 ± 1.65 | <0.001* |
| Osteopenia (n, %) | 2382 (31.67 %) | 3900 (30.06 %) | 0.041* |
| Osteoporosis (n, %) | 475 (6.32 %) | 928 (7.15 %) | 0.015* |

Continuous data are expressed as level ± standard deviation, and categorical data are expressed as number of patients (%). The *p*-values were calculated using the Chi-square test or Student *t* test.

Abbreviations: BMI, body mass index; FBS, fasting blood sugar; HbA1c, hemoglobin A1c; HDL, high density lipoprotein; MASLD, metabolic dysfunction-associated steatotic liver disease; TG, triglyceride; TyG, triglyceride-glucose index; WHR, waist to height ratio.

Supplementary Table S2

The distribution and association between genotypes of candidate SNPs in MASLD

| SNP | Genotype | MASLD | Non-MASLD | % | Odds ratio | 95 % CI | *p* |
| --- | --- | --- | --- | --- | --- | --- | --- |
| APOE- rs429358 |  |  |  |  |  |  |  |
| risk allele: T | C_C | 36 | 106 | 25.4 | 0.563 | 0.385-0.882 | 0.003 |
|  | C_T | 1092 | 2155 | 33.6 | 0.839 | 0.775-0.909 | <0.001 |
|  | T_T | 6159 | 10201 | 37.6 | Reference |  |  |
|  | Trend |  |  |  |  |  | <0.001 |
| PNPLA3- rs738409 |  |  |  |  |  |  |  |
| Risk allele: G | C_C | 2551 | 5002 | 33.8 | 0.878 | 0.824-0.936 | <0.001 |
|  | C_G | 3441 | 5927 | 36.7 | Reference |  |  |
|  | G_G | 1324 | 1591 | 45.4 | 1.433 | 1.318-1.560 | <0.001 |
|  | Trend |  |  |  |  |  | <0.001 |
| PNPLA3- rs2896019 |  |  |  |  |  |  |  |
| risk allele: G | G_G | 1374 | 1705 | 44.6 | 1.400 | 1.289-1.520 | <0.001 |
|  | G_T | 3456 | 6002 | 36.5 | Reference |  |  |
|  | T_T | 2480 | 4807 | 34.0 | 0.896 | 0.840-0.955 | 0.001 |
|  | Trend |  |  |  |  |  | <0.001 |
| GCKR- rs780094 |  |  |  |  |  |  |  |
| risk allele: T | C_C | 1853 | 3414 | 35.2 | 0.935 | 0.872-1.002 | 0.058 |
|  | C_T | 3658 | 6301 | 36.7 | Reference |  |  |
|  | T_T | 1805 | 2805 | 39.2 | 1.109 | 1.032-1.190 | 0.005 |
|  | Trend |  |  |  |  |  | 0.001 |
| GCKR- rs1260326 |  |  |  |  |  |  |  |
| risk allele: T | C_C | 1742 | 3256 | 34.9 | 0.926 | 0.863-0.994 | 0.035 |
|  | C_T | 3659 | 6335 | 36.6 | Reference |  |  |
|  | T_T | 1915 | 2929 | 39.5 | 1.133 | 1.055-1.215 | 0.001 |
|  | Trend |  |  |  |  |  | <0.001 |
| TM6SF2-rs58542926 |  |  |  |  |  |  |  |
| risk allele: T | C_C | 6260 | 10997 | 36.3 | Reference |  |  |
|  | C_T | 998 | 1456 | 40.7 | 1.204 | 1.105-1.313 | <0.001 |
|  | T_T | 43 | 46 | 48.3 | 1.642 | 1.082-2.492 | 0.019 |
|  | Trend |  |  |  |  |  | <0.001 |

Categorical data are expressed as number of patients. *p* value is analyzed by logistic regression. Reference was designated based on the most prevalent genotype. Abbreviations: APOE, Apolipoprotein E; CI, confidence interval; GCKR, Glucokinase regulator; MASLD, metabolic dysfunction-associated steatotic liver disease; PNPLA3, patatin like phospholipase domain containing 3; SNP, single nucleotide polymorphisms; TM6SF2: transmembrane 6 superfamily member 2.

Supplementary Table S3

Associated genotypes of SNPs in whole population with HU

| SNP | Genotype | HU | Non-HU | % | Odds ratio | 95 % CI | *p* |
| --- | --- | --- | --- | --- | --- | --- | --- |
| PNPLA3- rs738409 |  |  |  |  |  |  |  |
| Risk allele: C | C_C | 1773 | 5665 | 23.8 | 1.167 | 1.084-1.255 | <0.001 |
|  | C_G | 1954 | 7283 | 21.2 | Reference |  |  |
|  | G_G | 594 | 2286 | 20.6 | 0.968 | 0.873-1.074 | 0.543 |
|  | Trend |  |  |  |  |  | 0.001 |
| PNPLA3- rs2896019 |  |  |  |  |  |  |  |
| Risk allele: T | G_G | 618 | 2425 | 20.3 | 0.948 | 0.857-1.050 | 0.306 |
|  | G_T | 1971 | 7335 | 21.1 | Reference |  |  |
|  | T_T | 1729 | 5445 | 24.1 | 1.182 | 1.098-1.272 | <0.001 |
|  | Trend |  |  |  |  |  | <0.001 |
| GCKR- rs780094 |  |  |  |  |  |  |  |
| Risk: T | C_C | 1013 | 4188 | 19.5 | 0.841 | 0.774-0.914 | <0.001 |
|  | C_T | 2194 | 7628 | 22.3 | Reference |  |  |
|  | T_T | 1114 | 3418 | 24.6 | 1.134 | 1.043-1.230 | 0.003 |
|  | Trend |  |  |  |  |  | <0.001 |
| GCKR- rs1260326 |  |  |  |  |  |  |  |
| Risk: T | C_C | 955 | 3980 | 19.4 | 0.838 | 0.770-0.912 | <0.001 |
|  | C_T | 2195 | 7665 | 22.3 | Reference |  |  |
|  | T_T | 1171 | 3589 | 24.6 | 1.139 | 1.050-1.236 | 0.002 |
|  | Trend |  |  |  |  |  | <0.001 |

Categorical data are expressed as number of patients. *p* value is analyzed by logistic regression. Reference was designated based on the most prevalent genotype. Abbreviations: SNP, single nucleotide polymorphism; HU, hyperuricemia; CI, confidence interval; GCKR, Glucokinase regulator; PNPLA3, patatin like phospholipase domain containing 3.

Supplementary Table S4

Associated SNPs of low BMD in obese population with HU

| SNP | Genotype | Low BMD | Normal BMD | % | Odds ratio | 95 % CI | *p* |
| --- | --- | --- | --- | --- | --- | --- | --- |
| GCKR- rs780094 |  |  |  |  |  |  |  |
| Risk: T | C_C | 49 | 1040 | 4.6 | 0.669 | 0.479-0.935 | 0.013 |
|  | C_T | 139 | 1974 | 6.6 | - |  |  |
|  | T_T | 62 | 888 | 6.5 | 0.992 | 0.728-1.351 | 0.957 |
|  | Trend |  |  |  |  |  | 0.096 |
| GCKR- rs1260326 |  |  |  |  |  |  |  |
| Risk: T | C_C | 47 | 981 | 4.6 | 0.687 | 0.489-0.966 | 0.030 |
|  | C_T | 138 | 1980 | 6.5 | - |  |  |
|  | T_T | 65 | 941 | 6.4 | 0.997 | 0.731-1.344 | 0.954 |
|  | Trend |  |  |  |  |  | 0.079 |

Categorical data are expressed as number of patients. *p* value is analyzed by logistic regression. Reference was designated based on the most prevalent genotype. Abbreviations: BMD, bone mineral density; CI, confidence interval; GCKR, glucokinase regulator; HU, hyperuricemia; SNP, single nucleotide polymorphism.

Supplementary Table S5

Associated SNPs of occurrence of low BMD in elderly population with HU

| SNP | Genotype | Low BMD | Normal BMD | % | Odds ratio | 95 % CI | *p* |
| --- | --- | --- | --- | --- | --- | --- | --- |
| GCKR- rs780094 |  |  |  |  |  |  |  |
| Risk: T | C_C | 227 | 1436 | 13.7 | 0.926 | 0.782-1.097 | 0.374 |
|  | C_T | 496 | 2906 | 14.6 | - |  |  |
|  | T_T | 256 | 1431 | 15.2 | 1.048 | 0.890-1.234 | 0.573 |
|  | Trend |  |  |  |  |  | 0.210 |
| GCKR- rs1260326 |  |  |  |  |  |  |  |
| Risk: T | C_C | 207 | 1372 | 13.1 | 0.622 | 0.522-0.741 | 0.001 |
|  | C_T | 505 | 2802 | 15.3 | - |  |  |
|  | T_T | 267 | 1509 | 14.9 | 0.729 | 0.620-0.858 | 0.001 |
|  | Trend |  |  |  |  |  | 0.123 |

Categorical data are expressed as number of patients (%). p value is analyzed by logistic regression. Reference was designated based on the most prevalent genotype. Abbreviations: BMD, bone mineral density; CI, confidence interval; GCKR, glucokinase regulator; HU, hyperuricemia; SNP, single nucleotide polymorphism.

Supplementary Table S6

Associated genotypes of SNPs in general population with low BMD

| SNP | Genotype | Low BMD | Normal BMD | % | Odds ratio | 95 % CI | *p* |
| --- | --- | --- | --- | --- | --- | --- | --- |
|  |  |  |  |  |  |  |  |
| GCKR- rs780094 |  |  |  |  |  |  |  |
| Risk: T | C_C | 2034 | 3230 | 38.6 | 0.920 | 0.859-0.985 | 0.017 |
|  | C_T | 4045 | 5909 | 40.6 | Reference |  |  |
|  | T_T | 1906 | 2703 | 41.4 | 1.030 | 0.960-1.106 | 0.413 |
|  | Trend |  |  |  |  |  | 0.022 |
| GCKR- rs1260326 |  |  |  |  |  |  |  |
| Risk: T | C_C | 1932 | 3063 | 38.7 | 0.671 | 0.622-0.723 | <0.001 |
|  | C_T | 4035 | 5954 | 40.4 | Reference |  |  |
|  | T_T | 2018 | 2825 | 41.7 | 1.054 | 0.983-1.130 | 0.139 |
|  | Trend |  |  |  |  |  | 0.017 |
| PPARGC1A-rs8192678 |  |  |  |  |  |  |  |
| Risk: T | C_C | 2450 | 3868 | 38.8 | 0.924 | 0.866-0.986 | 0.017 |
|  | C_T | 3944 | 5755 | 40.7 | Reference |  |  |
|  | T_T | 1515 | 2133 | 41.5 | 1.036 | 0.960-1.120 | 0.365 |
|  | Trend |  |  |  |  |  | 0.012 |

Categorical data are expressed as number of patients. *p* value is analyzed by logistic regression. Reference was designated based on the most prevalent genotype. Abbreviations: BMD, bone mineral density; CI, confidence interval; GCKR, glucokinase regulator; PPARGC1A, peroxisome proliferator-activated receptor γ coactivator 1α; SNP, single nucleotide polymorphisms.

**Supplementary Table S7**

**The insulin resistance marker and associated SNPs of low BMD in non-obese general population with hyperuricemia**

|  | | Low BMD | | | | Normal BMD | | | *p* | |
| --- | --- | --- | --- | --- | --- | --- | --- | --- | --- | --- |
| TyG-WHR | | 7.36 ± 0.77 | | | | 7.24 ± 0.76 | | | <0.001 | |
| SNP | Genotype | | Low BMD | Normal BMD | % | | Odds ratio | 95 % CI | | *p* |
| GCKR- rs780094 |  | |  |  |  | |  |  | |  |
| Risk allele: T | C_C | | 336 | 532 | 38.7 | | 0.868 | 0.736-1.024 | | 0.190 |
|  | C_T | | 789 | 1094 | 41.9 | | Reference |  | |  |
|  | T_T | | 437 | 538 | 44.8 | | 1.116 | 0.955-1.304 | | 0.009 |
|  | Trend | |  |  |  | |  |  | | 0.008 |
| GCKR- rs1260326 |  | |  |  |  | |  |  | |  |
| Risk allele: T | C_C | | 318 | 504 | 38.7 | | 0.877 | 0.741-1.037 | | 0.067 |
|  | C_T | | 786 | 1092 | 41.9 | | Reference |  | |  |
|  | T_T | | 458 | 568 | 44.6 | | 1.120 | 0.961-1.306 | | 0.079 |
|  | Trend | |  |  |  | |  |  | | 0.010 |

Continuous data are expressed as level ± standard deviation. Categorical data are expressed as number of patients. *p* value is analyzed by logistic regression. Reference was designated based on the most prevalent genotype. Abbreviations: BMD, bone mineral density; CI, confidence interval; GCKR, glucokinase regulator; SNP, single nucleotide polymorphism; TyG-WHR, triglyceride-glucose index-waist to height ratio.

**Supplementary Table S8**

**The insulin resistance marker and associated SNPs of occurrence of low BMD in young general population with hyperuricemia**

|  | | Low BMD | | | Normal BMD | | | | *p* | |
| --- | --- | --- | --- | --- | --- | --- | --- | --- | --- | --- |
| TyG-WHR | | 7.29 ± 0.77 | | | 7.21 ± 0.76 | | | | < 0.001 | |
| SNP | Genotype | | Low BMD | Normal BMD | | % | Odds ratio | 95 % CI | | *p* |
| GCKR- rs780094 |  | |  |  | |  |  |  | |  |
| Risk allele: T | C_C | | 228 | 468 | | 32.8 | 0.961 | 0.794-1.163 | | 0.360 |
|  | C_T | | 513 | 1012 | | 33.6 | Reference |  | |  |
|  | T_T | | 308 | 476 | | 39.3 | 1.276 | 1.068-1.529 | | 0.007 |
|  | Trend | |  |  | |  |  |  | | 0.007 |
| GCKR- rs1260326 |  | |  |  | |  |  |  | |  |
| Risk allele: T | C_C | | 219 | 437 | | 33.0 | 0.972 | 0.747-1.112 | | 0.368 |
|  | C_T | | 508 | 1017 | | 33.3 | Reference |  | |  |
|  | T_T | | 322 | 502 | | 39.1 | 1.284 | 1.077-1.531 | | 0.005 |
|  | Trend | |  |  | |  |  |  | | 0.015 |

Continuous data are expressed as level ± standard deviation. Categorical data are expressed as number of patients. *p* value is analyzed by logistic regression. Reference was designated based on the most prevalent genotype. Abbreviations: BMD, bone mineral density; CI, confidence interval; GCKR, glucokinase regulator; SNP, single nucleotide polymorphism; TyG-WHR, triglyceride-glucose index-waist to height ratio.

Supplementary Table S9

Associated SNPs of less CMRFs with HU

| SNP | Genotype | HU | Non-HU | % | Odds ratio | 95 % CI | *p* |
| --- | --- | --- | --- | --- | --- | --- | --- |
| PNPLA3- rs2896019 |  |  |  |  |  |  |  |
| Risk: T | G_G | 196 | 560 | 25.9 | 0.930 | 0.766-1.129 | 0.463 |
|  | G_T | 469 | 1246 | 27.3 | Reference |  |  |
|  | T_T | 375 | 817 | 31.5 | 1.219 | 1.037-1.434 | 0.016 |
|  | Trend |  |  |  |  |  | 0.028 |

Categorical data are expressed as number of patients. *p* value is analyzed by logistic regression. Reference was designated based on the most prevalent genotype. Abbreviations: CMRF, metabolic syndrome and cardiometabolic risk factor; CI, confidence interval; HU, hyperuricemia; MASLD, metabolic dysfunction-associated steatotic liver disease; PNPLA3, patatin like phospholipase domain containing 3; SNP: single nucleotide polymorphism.

**Supplementary Table S10**

**The genetic effects of T alleles in GCKR- rs780094 and GCKR- rs1260326 with adjustment for gender, menopause and smoking**

| SNP | Risk allele | Frequency | | Adjusted for gender, menopause and smoking | | | |
| --- | --- | --- | --- | --- | --- | --- | --- |
|  |  | Low BMD | Normal BMD | | Odds ratio | 95 % CI | *p* |
| GCKR- rs780094 | T | 0.511 | 0.489 | | 1.040 | 1.010-1.071 | 0.008 |
| GCKR- rs1260326 | T | 0.511 | 0.489 | | 1.045 | 1.014-1.076 | 0.004 |

Abbreviations: BMD, bone mineral density; CI, confidence interval; GCKR, glucokinase regulator; SNP, single nucleotide polymorphism; TyG-WHR, triglyceride-glucose index-waist to height ratio.

**Supplementary Table 11.**

**The insulin resistance marker and associated SNPs of occurrence of low BMD in young MASLD with HU**

|  | | | Low BMD | | | | Normal BMD | | | *p* | |
| --- | --- | --- | --- | --- | --- | --- | --- | --- | --- | --- | --- |
| TyG-WHR | | | 7.56 ± 0.73 | | | | 7.47 ± 0.75 | | | < 0.001 | |
| SNP | Genotype | | Low BMD | Normal BMD | % | | Odds ratio | 95 % CI | | *p* | |
| GCKR- rs780094 |  | |  |  |  | |  |  | |  | |
| Risk: T | C_C | | 135 | 281 | 32.5 | | 0.913 | 0.713-1.170 | | 0.473 | |
|  | C_T | | 302 | 574 | 34.5 | | Reference |  | |  | |
|  | T_T | | 199 | 303 | 39.6 | | 1.248 | 0.996-1.566 | | 0.055 | |
|  | Trend | |  |  |  | |  |  | | 0.021 | |
| GCKR- rs1260326 |  | |  |  |  | |  |  | |  | |
| Risk: T | C_C | | 129 | 256 | 33.5 | | 0.974 | 0.757-1.255 | | 0.841 | |
|  | C_T | | 302 | 584 | 34.1 | | Reference |  | |  | |
|  | T_T | | 205 | 318 | 39.2 | | 1.247 | 0.997-1.259 | | 0.053 | |
|  | Trend | |  |  |  | |  |  | | 0.030 | |

Continuous data are expressed as level ± standard deviation. Categorical data are expressed as number of patients. *p* value is analyzed by logistic regression. Reference was designated based on the most prevalent genotype. Abbreviations: BMD, bone mineral density; CI, confidence interval; GCKR, glucokinase regulator; HU, hyperuricemia; SNP, single nucleotide polymorphism; TyG-WHR, triglyceride-glucose index-waist to height ratio.

**Supplementary Table 12.**

**The insulin resistance marker and associated SNPs of occurrence of low BMD in non-obese MASLD with HU**

|  | | | Low BMD | | | | Normal BMD | | | *p* | |
| --- | --- | --- | --- | --- | --- | --- | --- | --- | --- | --- | --- |
| TyG-WHR | | | 7.58 ± 0.72 | | | | 7.48 ± 0.75 | | | < 0.001 | |
| SNP | Genotype | | Low BMD | Normal BMD | % | | Odds ratio | 95 % CI | | *p* | |
| GCKR- rs780094 |  | |  |  |  | |  |  | |  | |
| Risk: T | C_C | | 185 | 297 | 38.4 | | 0.824 | 0.660-1.029 | | 0.088 | |
|  | C_T | | 433 | 573 | 43.0 | | Reference |  | |  | |
|  | T_T | | 270 | 314 | 46.2 | | 1.138 | 0.927-1.397 | | 0.118 | |
|  | Trend | |  |  |  | |  |  | | 0.010 | |
| GCKR- rs1260326 |  | |  |  |  | |  |  | |  | |
| Risk: T | C_C | | 176 | 277 | 38.9 | | 0.856 | 0.683-1.074 | | 0.099 | |
|  | C_T | | 429 | 578 | 42.6 | | Reference |  | |  | |
|  | T_T | | 283 | 329 | 46.2 | | 1.159 | 0.947-1.419 | | 0.084 | |
|  | Trend | |  |  |  | |  |  | | 0.016 | |

Continuous data are expressed as level ± standard deviation. Categorical data are expressed as number of patients. *p* value is analyzed by logistic regression. Reference was designated based on the most prevalent genotype. Abbreviations: BMD, bone mineral density; CI, confidence interval; GCKR, glucokinase regulator; HU, hyperuricemia; SNP, single nucleotide polymorphism; TyG-WHR, triglyceride-glucose index-waist to height ratio.
